# Comparison of Clinical and Radiological Outcomes of Total Knee Arthroplasty in Osteoarthritic Patients

**DOI:** 10.7759/cureus.60933

**Published:** 2024-05-23

**Authors:** Sukhjinder Singh, Harry Mehta, Saryu Gupta, Jaspreet Singh, Amandeep S Bakshi

**Affiliations:** 1 Orthopaedics, Government Medical College, Patiala, Patiala, IND; 2 Radiodiagnosis, Government Medical College, Patiala, Patiala, IND; 3 Radiodiagnosis, Rajindra Hospital, Patiala, IND

**Keywords:** knee society score, posterior tibial slope, medial proximal tibial angle, distal femoral cut angle, total knee replacement (tkr)

## Abstract

Background

The knee is the joint most commonly affected by osteoarthritis, more than any other. Osteoarthritis is a progressive, long-term condition that leads to the deterioration of joint tissue and cartilage, resulting in pain and impairment. Total knee arthroplasty (TKA) is a successful intervention that improves functional capability, decreases pain, and enhances quality of life. We conducted this study to evaluate whether radiological parameters following TKA influence the clinical outcomes of patients with knee osteoarthritis.

Methods

The study was conducted on patients treated for knee osteoarthritis at the Department of Orthopedics, Rajindra Hospital and Government Medical College, Patiala, Punjab, in collaboration with the Department of Radiology over a period of 1.5 years. A total of 152 patients diagnosed with knee osteoarthritis were included in the study; all underwent TKA. Patients underwent clinical evaluation and were graded using the Knee Society Score (KSS) during follow-up examinations. Pain was evaluated using the Visual Analog Scale (VAS). Postoperative X-rays were obtained, and various angles, including the distal femoral angle (DFA), the proximal tibial angle (PTA), and the posterior slope angle (PSA), were measured. Patient follow-up was conducted at three days, three months, and six months. Subsequently, a comparison of the clinical and radiological outcomes of TKA was performed.

Results

In this study, a total of 152 patients participated, with the majority falling into the 61-70 age group. Of these patients, 40.13% were female and 59.87% were male. The average medial DFA was 94.05°, the average medial PTA was 89.31°, and the PSA was 6.6°. Patients with a medial DFA of 94.05° (±3), a medial PTA of 89.31° (±3), and a PSA of 6.6° (±3) were categorized into the normal group.

Conclusion

Patients with DFA, PTA, and PSA in the normal range demonstrate improved KSS and clinical outcomes.

## Introduction

Osteoarthritis (OA) of the knee is the most prevalent cause of knee discomfort in the elderly [1]. For patients experiencing severe pain due to end-stage knee arthritis, total knee replacement (TKR) surgery is an effective method for reducing pain and improving both function and quality-adjusted life years [2]. Currently, total knee arthroplasty (TKA) is considered the optimal treatment option for patients experiencing osteoarthritic knee pain, particularly when conservative treatment measures have been unsuccessful [3]. TKA may also be indicated in cases where there is significant deformity at the knee, such as malalignment of the mechanical, anatomical, or kinematic axis, with evidence of OA [4]. The primary advantage of TKA lies in the restoration of the mechanical axis with a stable prosthesis, achieved through both bone resection and soft tissue balancing. The outcomes of the procedure, patient satisfaction, and the improved longevity of the implant since its introduction, have made this procedure widely accepted for affording relief of pain, restoration of range of motion (ROM), stability, and function.

Some concerns have arisen regarding the surgery, particularly concerning the fitting of the prosthesis, which is one of the most critical aspects to be addressed during TKA [5]. Previous studies have indicated that implant malposition can lead to postoperative malalignment of the limb, which may result in poor outcomes and decreased longevity of the prosthesis [6-8]. The Knee Society Score (KSS) system assesses the knee both clinically and functionally to provide a comprehensive assessment of the knee joint and aid in evaluating the functional outcome [9]. In the mechanical axis (anteroposterior view), the medial distal femoral angle (MDFA) and medial proximal tibial angle (MPTA) are measured; these should normally be 95° and 90°, respectively [10]. In the sagittal plane (lateral view), another radiological parameter used to evaluate the outcome of TKA is the posterior tibial slope angle (PSA) [11], which is the angle formed between the vertical line of the tibial anatomical axis and the tibial plateau tangent [12]. PSA reflects the tilt of the tibial plateau and plays an important role in knee joint stability and biomechanics. PSA varies significantly depending on the reference axis used in measurement. A normal PSA in a post-TKR X-ray is 5 degrees.

We conducted this study to evaluate whether radiological parameters following TKA influence the clinical outcomes of patients with knee OA. A total of 152 patients diagnosed with knee joint OA were included in the study, all of whom underwent TKA.

## Materials and methods

This was a prospective study conducted on patients undergoing TKR at the Department of Orthopedics, Rajindra Hospital and Government Medical College, Patiala, Punjab, in collaboration with the Department of Radiology for 1.5 years. Patient follow-up was conducted at three days, three months, and six months. Inclusion criteria included patients with OA of the knee undergoing TKA. Exclusion criteria included patients with a preexisting muscular or neurological disease, rheumatoid arthritis, or previous knee surgeries other than arthroscopy.

The objective of this study was to evaluate the influence of specific radiological parameters, namely, the DFA, PTA, and PSA, on the clinical outcomes of patients with knee OA following TKA.

Written informed consent was obtained from each patient enrolled in the study. Patients were evaluated both clinically and radiologically. After undergoing total knee arthroplasty, follow-up was conducted on postoperative day 3, at three months, and at six months. During the follow-up examinations, patients were clinically evaluated for pain and function. Pain assessment was performed using the Visual Analog Scale (VAS): this self-reported measurement of symptoms ranged from 'no pain' (0) at the left end of the scale to 'worst pain' (10) at the right end. Functional outcomes were assessed using the KSS. Radiological measurements were performed using standardized techniques. The DFA was measured as the angle between the femoral mechanical axis and the distal femoral condyle line. The PTA was measured as the angle between the tibial mechanical axis and the proximal tibial plateau. The PSA was measured as the angle between the vertical line of the tibial anatomical axis and the tibial plateau tangent on lateral radiographs. Data and statistical analysis were performed using Microsoft Excel.

This study was approved by the ethics committee of our hospital (Institutional Ethics Committee, Government Medical College, Patiala) with approval number 30144. All enrolled patients provided consent for participation in this study before enrollment.

## Results

**Figure 1:**
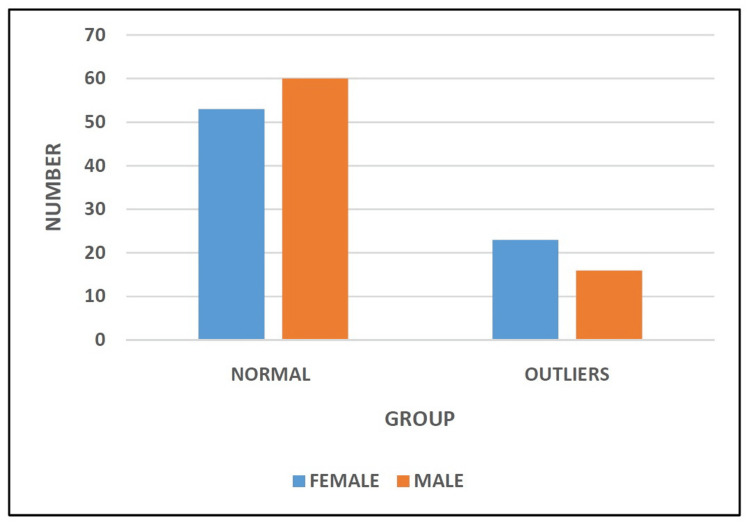
Distribution of study participants based on gender.

In this study, there were a total of 152 patients, of which 76 were males and 76 were females. We divided our patients into two groups based on inliers and outliers. In the Normal Group, there were 53 females and 60 males, while in the Outliers Group, there were 23 females and 16 males (Table 1 and Figure 1).

**Table 1:** Distribution of study participants based on gender.

Gender	Normal		Outliers		Total	
	Number	Percentage	Number	Percentage	Number	Percentage
Female	53	46.9%	23	59.0%	76	50.0%
Male	60	53.1%	16	41.0%	76	50.0%
Total	113	100%	39	100%	152	100%

**Figure 2:**
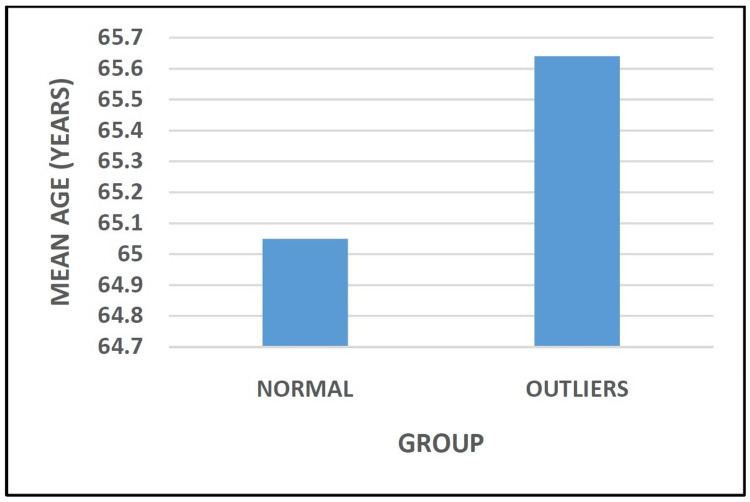
Distribution of study participants based on age.

The mean age of the patients in the Normal Group and the Outliers Group was 65.05 ± 3.02 years and 65.64 ± 2.92 years, respectively (Figure 2 and Table 2).

**Table 2:** Distribution of study participants based on age.

Age	Mean ± Std. Dev.	P-value
Normal (N=113)	65.05 ± 3.02	0.272
Outliers (N=39)	65.64 ± 2.92	

**Figure 3:**
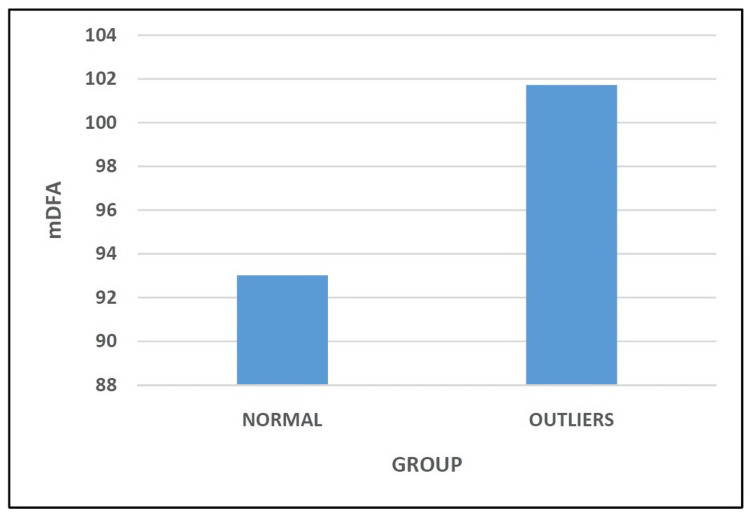
Comparison of medial distal femoral angle.

The mDFA of the patients in the Normal Group and the Outliers Group was 93.02° ± 0.71 and 101.72° ± 1.85, respectively (Figure 3 and Table 3). Statistical analysis revealed this comparison between the two groups to be significant (p < 0.05).

**Table 3:** Comparison of medial distal femoral angle.

Medial Distal Femoral Angle	Mean ± Std. Dev.	P-value
Normal (N=113)	93.02° ± 0.71	0.000
Outliers (N=39)	101.72° ± 1.85	

**Figure 4:**
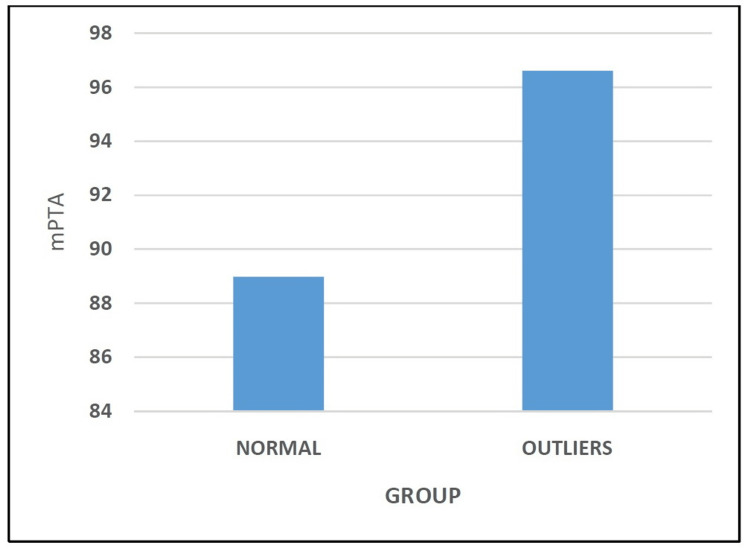
COMPARISON OF medial proximal tibial angle

The mPTA of the patients in the Normal Group and the Outliers Group was 88.98° ± 0.65 and 96.61° ± 1.25, respectively (Table 4 and Figure 4). Statistical analysis showed this comparison between the two groups to be significant (p < 0.05).

**Table 4:** Comparison of medial proximal tibial angle.

Medial proximal tibial angle	Mean ± Std. Dev.	P-value
Normal (N=113)	88.98° ± 0.65	0.000
Outliers (N=39)	96.61° ± 1.25	

**Figure 5:**
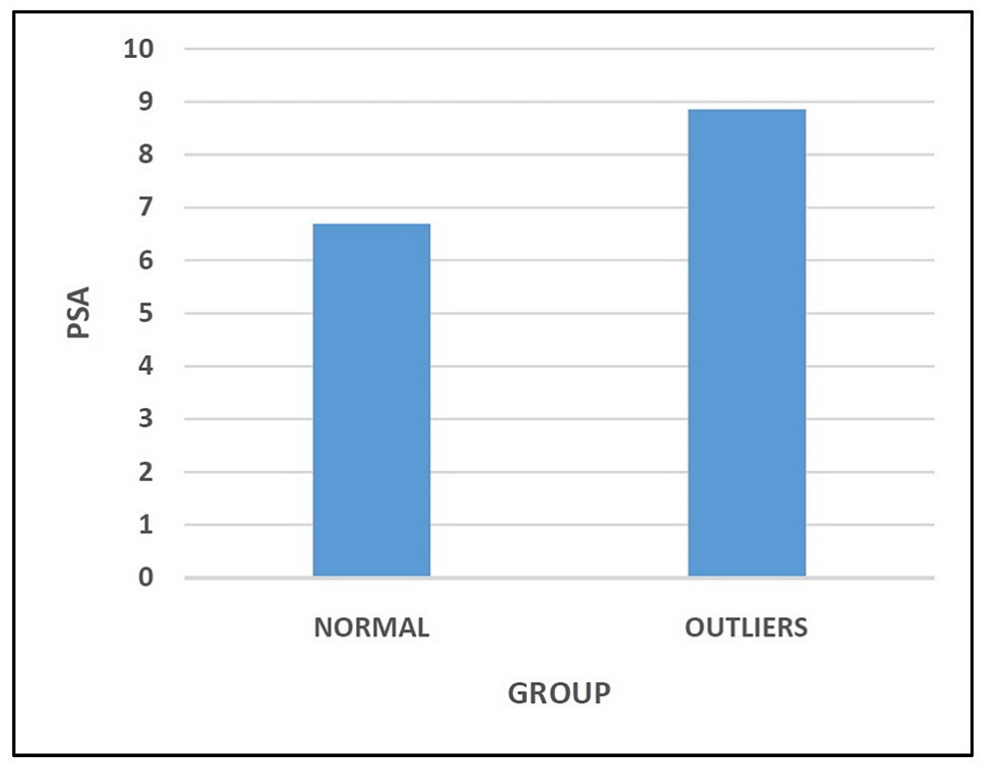
Comparison of posterior slope angle.

The PSA of the patients in the Normal Group and the Outliers Group was 6.69° ± 0.46 and 8.86° ± 0.70, respectively (Table 5 and Figure 5). Statistical analysis indicated that this comparison between the two groups was significant (p < 0.05).

**Table 5:** Comparison of posterior slope angle.

Posterior Slope Angle	Mean ± Std. Dev.	P-value
Normal (N=113)	6.69° ± 0.46	0.000
Outliers (N=39)	8.86° ± 0.70	

**Figure 6:**
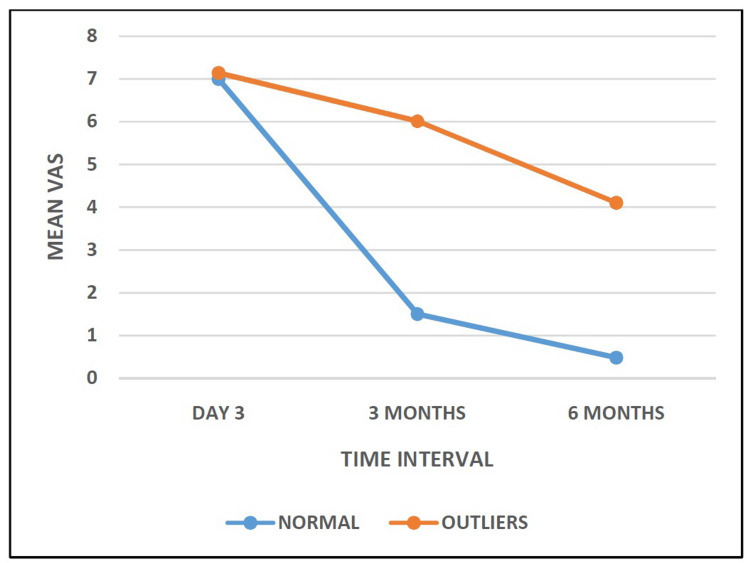
Showing comparison of Visual Analogue score in group A and group B.

The mean VAS score of patients in the Normal Group at day 3, at three months, and at six months were 7.00 ± 0.66, 1.50 ± 0.21, and 0.48 ± 0.15, respectively. The mean VAS scores for patients in the Outlier Group at day 3, at three months, and at six months were 7.14 ± 0.19, 6.01 ± 0.77, and 4.10 ± 0.64, respectively. Statistical analysis showed that the comparison between both groups was not significant (p > 0.05) at day 3; however, it was significant at three months and at six months (p < 0.05) (Table 6, Figure 6).

**Table 6:** Comparison of Visual Analogue Scale score at different intervals. VAS: Visual Analogue Scale.

Pain (VAS)	Normal (N=113)	Outliers (N=39)	P-value
Day 3	7.00 ± 0.66	7.14 ± 0.19	0.074
3 months	1.50 ± 0.21	6.01 ± 0.77	0.000
6 months	0.48 ± 0.15	4.10 ± 0.64	0.000

**Figure 7:**
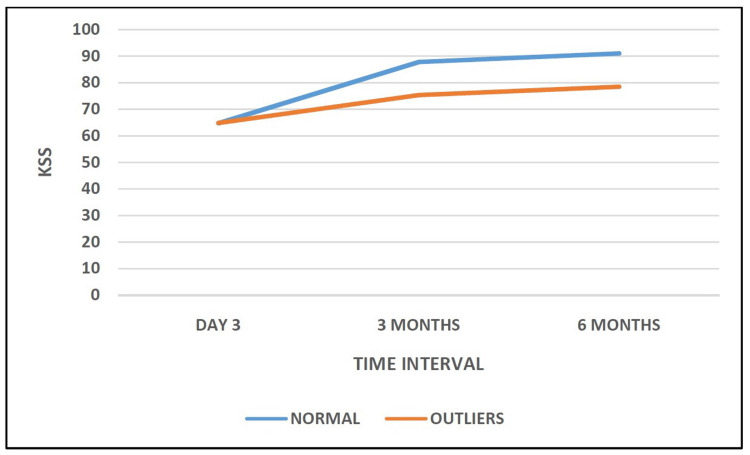
Comparison of Knee Society Scores in group A and group B.

The mean KSS of patients in the Normal Group at day 3, at three months, and at six months were 64.69 ± 0.41, 87.79 ± 0.49, and 91.03 ± 0.62, respectively. The mean KSS for patients in the Outlier Group at day 3, at three months, and at six months were 64.85 ± 0.54, 75.34 ± 2.22, and 78.50 ± 2.14, respectively. Statistical analysis showed that the comparison between both groups was not significant (p > 0.05) at day 3; however, it was significant at three months and at six months (p < 0.05) (Table 7, Figure 7).

**Table 7:** Comparison of Knee Society Score at different intervals.

KSS	Normal (N=113)	Outliers (N=39)	P-value
Day 3	64.69 ± 0.41	64.85 ± 0.54	0.109
3 months	87.79 ± 0.49	75.34 ± 2.22	0.000
6 months	91.03 ± 0.62	78.50 ± 2.14	0.000

## Discussion

There is limited data in the literature comparing the functional outcomes of TKA with postoperative radiological angles (DFA, PTA, and PSA). This study reinforces the positive correlation between the functional outcomes of TKA and these postoperative radiological angles. Upon comparing both groups, we discovered that patients in Group A, exhibiting normal radiological parameters (medial DFA, medial PTA, and PSA), demonstrated better VAS scores (p-value < 0.0005) and KSS (p-value < 0.0005) than patients in Group B at three months and six months.

In the current study, the mean age of patients was 64.2 in the inlier group and 67.3 in the outlier group. Our observation of the mean age aligns with findings from studies conducted by Kazarian GS et al., Charaya H et al., and Jose RS and Kannan V. From our study, we concluded that patients in the inlier group, who have medial DFA, medial PTA, and PSA within the normal range, exhibit a lower VAS score and higher KSS than patients in the outlier group, whose DFA, PTA, and PSA fall outside the normal range, as observed during the follow-up of postoperative TKR patients at three days, three months, and six months. Kazarian GS et al. (2021) conducted a similar study involving 262 patients. They found that patients with DFA, PTA, and PSA outliers were dissatisfied with their degree of pain relief compared to those with normal DFA, PTA, and PSA [13]. Our findings corroborate the results of the study conducted by Kazarian GS et al. Jose RS and Kannan V also conducted a similar prospective study on 20 patients with OA knees, in which they evaluated the functional outcome of TKR using radiographic alignment [14]. They discovered that the correct positioning of TKR components axially and rationally improves both functional and radiological outcomes. Our study, conducted on 152 patients, further reinforces this finding, indicating that postoperative TKR patients with radiographic DFA, PTA, and PSA within the normal range demonstrate superior functional outcomes compared to patients in the outlier group. Charaya et al. conducted a comparable study involving 60 knees in 42 patients with OA of the knee joint. They concluded that mild mechanical axis malalignment had no effect on functional outcomes following TKR in the short term [15]. Although we did not use the mechanical axis as a parameter for radiological evaluation, our results did not align with their findings based on medial DFA, PTA, and PSA.

One of the primary strengths of this study is the simultaneous evaluation of clinical and radiological parameters, allowing for a comprehensive assessment of therapeutic outcomes. Both physical and functional measures were included, providing a holistic view of the patients' progress. Additionally, the study's prospective design adds to its robustness. Limitations include (1) The relatively small sample size, which may affect the generalizability of the findings. Future studies with larger cohorts are needed to confirm these results; and (2) The study did not account for other potential confounding factors such as varying surgical techniques or postoperative rehabilitation protocols, which could influence the outcomes.

## Conclusions

Our study demonstrates a clear positive correlation between normal postoperative radiological parameters (medial DFA, PTA, and PSA) and improved functional outcomes in patients undergoing TKA. Patients with these parameters within the normal range exhibited significantly better pain relief and higher KSS at three and six months postoperatively. While the study carefully controlled for age and baseline functional status, future research should consider additional confounding factors such as surgical technique and rehabilitation protocols. These findings underscore the importance of achieving optimal radiological alignment during TKA to enhance patient outcomes. Larger, multi-center studies with long-term follow-up are recommended to further validate and expand upon these results.

Therefore, ensuring that DFA, PTA, and PSA are within the normal range in every case of TKA is imperative for improving its long-term functional outcomes.

## Figures and Tables

**Figure 8:**
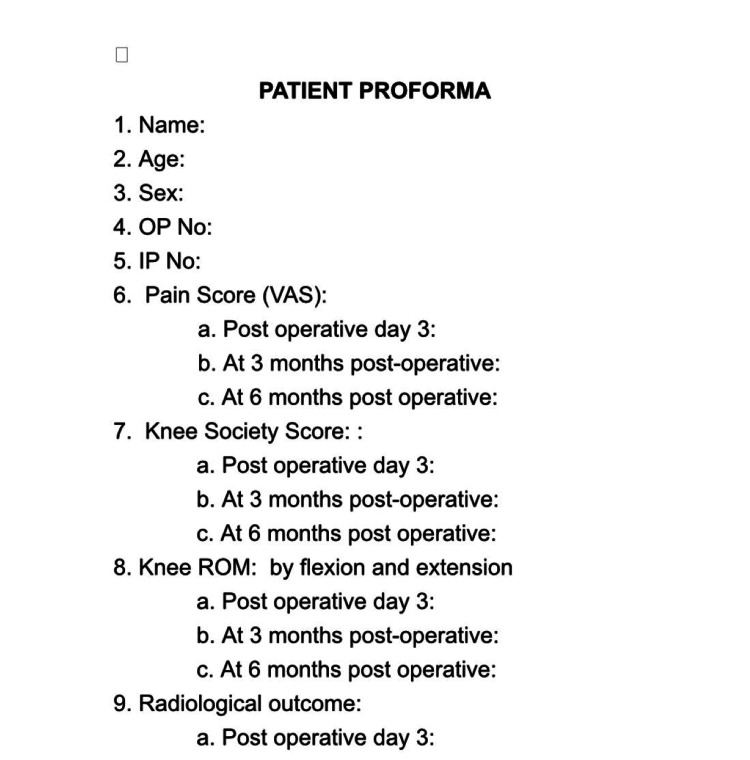
Patient Performa.

**Figure 9:**
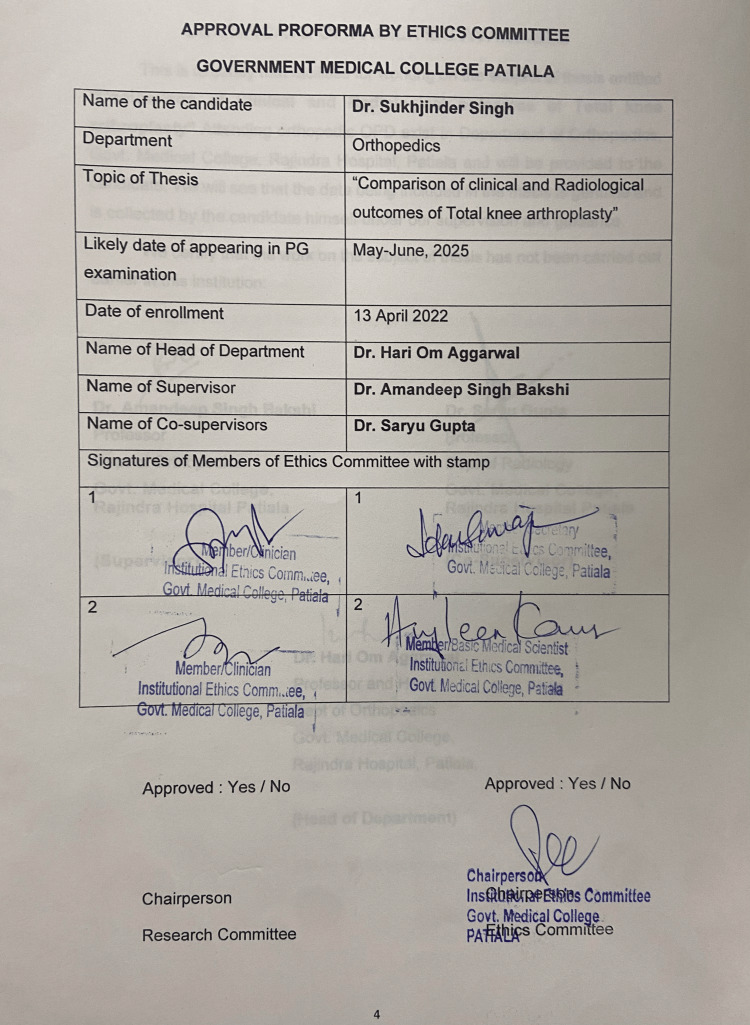
Ethics committee approval letter.

## Appendices

Appendix 1

**Table 8:** Date of assessment.

Clinical Assessment	Date
Day 3 (Post operative)	As per patient surgery
3 months (Post operative)	As per patient surgery
6 months (Post operative)	As per patient surgery

Appendix 2 

 Appendix 3                                                                      
